# Characterization of the development of the high-acuity area of the chick retina

**DOI:** 10.1016/j.ydbio.2024.03.005

**Published:** 2024-03-26

**Authors:** Jiho Choi, Heer V. Joisher, Hasreet K. Gill, Lucas Lin, Constance Cepko

**Affiliations:** 1 Department of Genetics, Blavatnik Institute; 2 Department of Ophthalmology, Harvard Medical School, United States; 3 Howard Hughes Medical Institute, United States; 4 These authors contributed equally to this work.

## Abstract

The fovea is a small region within the central retina that is responsible for our high acuity daylight vision. Chickens also have a high acuity area (HAA), and are one of the few species that enables studies of the mechanisms of HAA development, due to accessible embryonic tissue and methods to readily perturb gene expression. To enable such studies, we characterized the development of the chick HAA using single molecule fluorescent *in situ* hybridization (smFISH), along with more classical methods. We found that *Fgf8* provides a molecular marker for the HAA throughout development and into adult stages, allowing studies of the cellular composition of this area over time. The radial dimension of the ganglion cell layer (GCL) was seen to be the greatest at the HAA throughout development, beginning during the period of neurogenesis, suggesting that genesis, rather than cell death, creates a higher level of retinal ganglion cells (RGCs) in this area. In contrast, the HAA acquired its characteristic high density of cone photoreceptors posthatching, which is well after the period of neurogenesis. We also confirmed that rod photoreceptors are not present in the HAA. Analyses of cell death in the developing photoreceptor layer, where rods would reside, did not show apoptotic cells, suggesting that lack of genesis, rather than death, created the “rod-free zone” (RFZ). Quantification of each cone photoreceptor subtype showed an ordered mosaic of most cone subtypes. The changes in cellular densities and cell subtypes between the developing and mature HAA provide some answers to the overarching strategy used by the retina to create this area and provide a framework for future studies of the mechanisms underlying its formation.

## Graphical Abstract

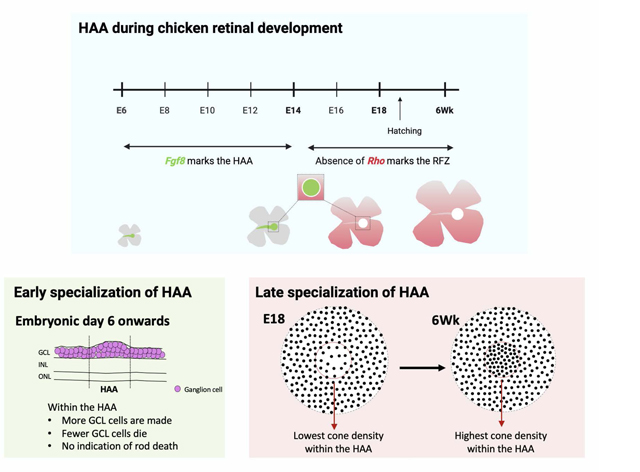

## INTRODUCTION

Humans, as well as some other primates, enjoy high acuity daylight vision. The region of the retina that endows us with this trait is referred to as the fovea (Latin:pit). The retinal cells in this area have a distinctive arrangement and composition (Kolb et al. 2020). While almost all mammalian retinas have rods, the photoreceptor type that captures and processes light in dim light conditions, as the majority of photoreceptors, the central portion of the fovea, the foveola, is devoid of rods. Instead, it is packed with cones, the photoreceptor type that initiates vision in the bright light conditions of the day. Cones are also the cell type which enable color vision, as cones can express different opsin proteins, each of which is tuned to capture light most effectively at different wavelengths. Other differences between the fovea and the surrounding retina include a lower ratio of cones to the output neurons of the retina, the RGCs (Bringmann et al. 2018). RGCs receive the results of the information transformations that are carried out by local retinal circuits, and pass this information on to multiple locations in the brain. The ratio of cones to RGCs in the fovea can be one-to-three, while outside of the fovea, there is convergence of signals emanating from many photoreceptors onto a single RGC. In addition, the cones in the primate fovea are more slender and densely packed than the peripheral cones. The interneurons of the fovea also differ from those in the periphery and are pushed aside during foveal development to create the foveal pit (Bringmann et al., 2018; Provis et al. 2013). All these adaptations lead to an increase in acuity in this area. As one ages, the chance of losing high acuity vision due to degeneration of the central retina increases. The fovea is located within the central retina, in an area designated as the macula, which is prone to degeneration, e.g., in age-related macular degeneration (AMD) (Fleckenstein et al. 2021).

Although retinal development has been well studied, we know very little about the mechanisms that govern development of the fovea, or of any HAA. In humans and some non-human primates, the work of Mann, Hendrickson, Provis and colleagues has provided foundational information on the development of the HAA (Mann 1964 Hendrickson 1992; Hendrickson and Kupfer 1976; Hendrickson and Yuodelis 1984; van Driel, Provis, and Billson 1990; Diaz-Araya and Provis 1992). In addition, mutations that affect the human fovea have been identified (Kuht et al 2022). However, due to the lack of a HAA in the typical model organisms, such as mice and zebrafish, the molecular and genetic aspects, even of these disease genes, remain relatively under-studied. The canine area centralis, defined as an area of high RGC density, exhibits a high cone density (Mowat et al. 2008; Beltran et al. 2014; Pichard et al. 2016). Interestingly, this area also shows vulnerability to human AMD genes, suggesting that it might serve to model human macular diseases. However, limited genetic tools and access hinder developmental studies in dogs. Human retinal organoids would seem to offer a system for discovery of such mechanisms. However, so far, foveal development has not been reported in organoid cultures (Cowan et al. 2020; Meyer et al. 2011; Nakano et al. 2012). Moreover, human organoids are expensive to culture, are slow to develop, and require constant tending.

Some birds and lizards have high acuity vision, with birds of prey having exceptionally high acuity vision. Some species even have two foveas, e.g., raptors and brown anoles (Rasys et al. 2021; Potier et al. 2017; Sannan et al. 2018). We discovered that chicks have the features of a HAA. Initially, we found that there is a central area with no rods, the RFZ (Bruhn and Cepko 1996). Further investigation showed that this area lines up with an area of high RGC density (da Silva and Cepko 2017), and a structure, the aster, originally reported by Morris to have an unusual arrangement of interneurons (Morris 1982). High RGC density is a feature used to define an area of highest acuity in many species (Hughes 1977), even those that do not have what is considered “high acuity” vision relative to e.g., humans. Since the chick does not have a pit, we refer to this area as a HAA. We have been investigating some of the gene expression patterns that govern HAA development in the chick. As reported previously in several species (McCaffery and Drager 1995), retinoic acid (RA) synthetic and degradative enzymes exhibit expression patterns suggestive of a role in the formation of this area. Similarly, *Fgf8* is expressed in an area suggestive of the HAA anlagen at embryonic stages (da Silva and Cepko, 2017). As chick embryos are amenable to perturbations, we were able to show that lack of RA signaling, and presence of *Fgf8*, were required to form this area. Degradation of RA in the developing human fovea also may occur, as we found that *Cyp26a1*, a degradative enzyme for RA, was localized to this area at fetal weeks 8–10, a time when retinal patterning is likely happening (da Silva and Cepko 2017). Interestingly, specific expression of this gene is also reported in the progenitors of primate fovea, as well as in foveal Mueller glia, in the adult human retina (Cowan et al. 2020). *Fabp5*, another protein that can regulate RA signaling, has this same expression pattern (Krueger et al. 2023; Voigt et al. 2021).

Several overarching mechanisms can account for development of the specific cellular features of a HAA e.g. differential genesis and/or cell death resulting in distinctive cellular compositions. Migration of cells in or out of this area, and/or differential expansion and contraction, can account for some of the cellular as well as morphological aspects (Diaz-Araya and Provis 1992; Hendrickson 1999; Springer 1999; Springer and Hendrickson 2004; Rasys et al. 2021). In order to begin to address such mechanisms, we undertook an investigation of the morphological and cellular aspects of development of the chick HAA.

## RESULTS

### Visualization of the HAA during chick retinal development

**Figure 1.**
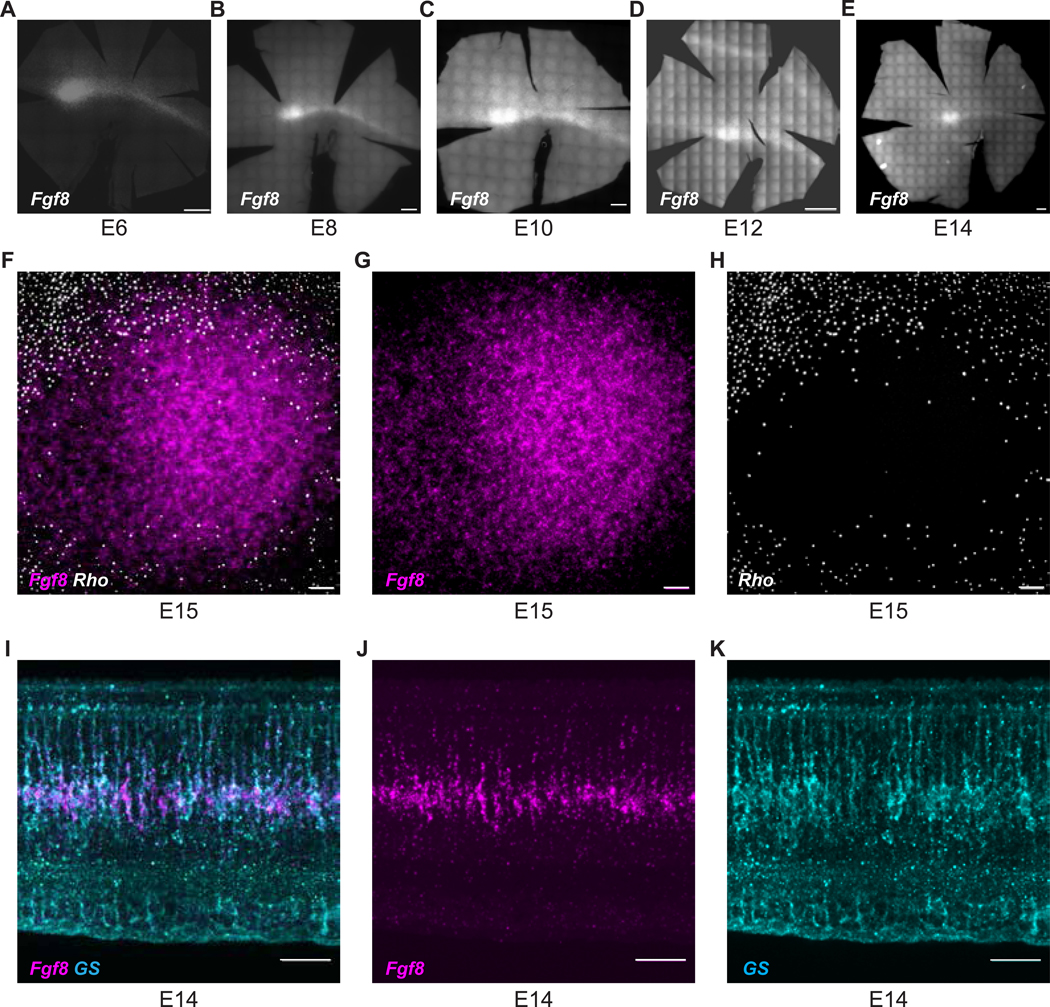
Visualization of the HAA during chick retinal development. **(A-E)** HCR *in situ* hybridization for *Fgf8* RNA on (A) E6, (B) E8, (C) E10, (D) E12 and (E) E14 retinal whole-mounts. Scale bars, 500 μm in all. **(F-H)** HAA visualized using multiplexed HCR *in situ* hybridization for *Fgf8* RNA (G) and *Rho* RNA (H) on a E15 retinal whole-mount. Scale bar, 50 μm. **(I-K)** E14 retinal cross-section through the HAA with HCR *in situ* hybridization for *Fgf8* RNA (J) and immunofluorescence staining for Glutamine Synthetase (GS) (K). Scale bar, 25 μm. D, Dorsal; V, Ventral.

To study the development of the HAA, it is critical to locate it during retinal development. Unlike foveas, the chick HAA lacks a steep decline in retinal thickness_,_ or “pit” (Morris 1982), which is an anatomical feature used to identify the HAA in humans and other primates (Hendrickson 1992). Even if the chick had such a feature, it likely would not be obvious at early developmental stages, as it is not evident until relatively late in development in primates (Springer and Hendrickson 2004). In the later stages of chick retinal development (embryonic day 15 (E15) onwards), the HAA can be detected by the absence of rhodopsin (*Rho*), the photopigment specific to rod photoreceptors (Bruhn and Cepko 1996). We previously showed that *Fgf8* is expressed in the area of the developing HAA during embryonic day 4–6 (E4 to E6) (da Silva and Cepko 2017), but we did not show that it marked this same area throughout development. In late stages of chick development, single-cell RNA sequencing (scRNA-seq) showed *Fgf8* expression in the central retina (Yamagata, Yan, and Sanes 2021). To determine if *Fgf8* marks the developing HAA throughout development, the spatiotemporal expression patterns of *Fgf8* were examined on retinal whole-mounts (E6 to E15) using the *in situ* HCR technique (Choi et al. 2018). As we saw previously, *Fgf8* was enriched in a naso-central spot, along with a temporally extended streak at E6 (da Silva and Cepko 2017). From E6 on, *Fgf8* expression exhibited a naso-central spot and nasal-temporal stripe that was diffuse on the dorsal side, but quite sharp on the ventral side (Fig. 1A–E). Faint *Fgf8* expression was detected ventrally starting from E10, which became more noticeable around E12. As rods are absent from the HAA, but the rod marker, *Rho*, does not express until E15, we used E15 whole mounts to investigate if the naso-central spot of *Fgf8* aligned with the RFZ. The naso-central spot of *Fgf8* expression seen throughout chick retinal development overlapped perfectly with the RFZ (Fig. 1F–H). In terms of the cell type(s) that express Fgf8, several lines of evidence suggest that retinal progenitor cells (RPCs) express it. Vogel-Hopper et al. (2000) and Martinez-Morales et al. (2005) reported it present in the chick central retina at the optic vesicle stage, prior to the formation of any neurons. We isolated E6 chick *Fgf8*^+^ cells using ProbeSeq, a method that uses ISH signals for cell isolation by FACS (Amamoto et al. 2019). Genes that were differentially expressed between *Fgf8*^+^ and *Fgf8*^−^ cells were enriched for early differentiation markers in the *Fgf8*^−^ population suggesting that the *Fgf8*^+^ cells were non-differentiated cells, or RPCs. Furthermore, the morphology of the early *Fgf8*^+^ cells suggests that they are RPCs. Vogel-Hopper et al. (2000) suggested that more mature retinas expressed *Fgf8* in Mueller glia, according to the location and morphology of *Fgf8*^+^ cells. We further investigated the cell type that expresses *Fgf8* later during development using E14 retinal sections stained for glutamine synthetase (GS), a specific Mueller glial gene. The *Fgf8*^+^ cells in the HAA were seen to co-express GS later (Fig. 1I–K). As Mueller glia retain expression of RPC genes (Blackshaw et al. 2004), this result also suggested expression in RPCs. Taken together, these data demonstrate that the naso-central spot of *Fgf8* expression persistently marks the HAA during embryonic retinal development and strongly suggest that *Fgf8* is expressed within specific RPCs early, and Mueller glia later.

### Morphological development of the HAA

**Figure 2.**
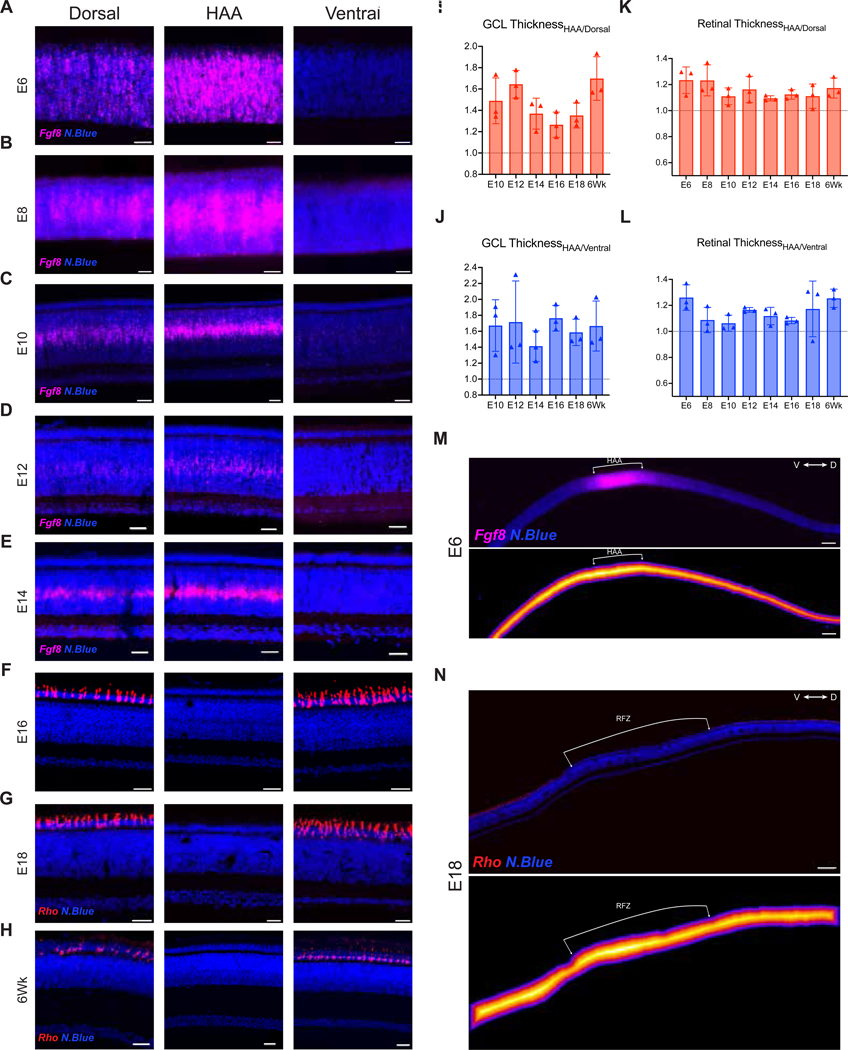
Morphological development of the HAA. **(A-H)** Retinal cross-sections of the HAA and neighboring dorsal and ventral regions (750μm away from the HAA) using E6 to 6Wks retinas. HAA was visualized using HCR *in situ* hybridization for *Fgf8* RNA on E6 to E14 retinas (A-E), and for *Rho* RNA on E16, E18 and 6Wks retinas (F-H). Dorsal-HAA-Ventral sections were further stained with NucBlue to visualize retinal cell layers. Scale bar, 25 μm. **(I-L)** Bar graph comparing intra-retinal GCL thickness ratios (I) HAA/dorsal region (J) HAA/ventral region and total retinal thickness ratios (K) HAA/dorsal region (L) HAA/ventral region during chick retinal development. **(M, N)** Total retinal thickness distance maps for retinal cross-sections of the HAA and neighboring dorsal and ventral regions from E6 (I) and E18 (J) retinas. The HAA was visualized using HCR *in situ* hybridization for *Fgf8* RNA on E6 retina, and for *Rho* RNA on an E18 retina. Areas depicted in red or brighter colors correspond to regions with greatest distance from the center to the edge, while those in blue or darker shades represent smaller distance from the center to the edge along the radial axis. Scale bar, 100 μm. GCL, Ganglion cell layer.

We examined the cellular and morphological aspects of the developing HAA over time using the *Fgf8* and *Rho* HCR signal in conjunction with measurements of retinal layers and cell subtypes. The area of highest acuity for many species has been defined as the area with the highest density of RGCs, located within the GCL (Curcio et al. 1990; Querubin et al. 2009). In keeping with this, we previously reported that the GCL was thickest (i.e., radial dimension in cross-section) within the HAA at E18, with a ~1.6-fold higher cell density compared to neighboring regions (da Silva and Cepko 2017). To investigate the morphological development of the GCL within the HAA, the retinal cell layers from embryonic and posthatch retinas (E6 to 6Wks) were visualized by staining dorso-ventral cross sections that spanned the HAA using a nuclear dye (Fig. 2A–H) The individual cellular and plexiform layers in the chick retina start to be distinct at E8, with continued sharpening of their borders throughout development (Doh et al. 2010). The thickness of the specific retinal layers was quantified by identifying the boundaries of individual layers and measuring the distance between them along the radial axis on the retinal cross sections. The GCL was observed to be the thickest within the HAA when compared to its neighboring dorsal and ventral regions, with p-values of 2.4*10^−7^ and 9.5*10^−7^ respectively, throughout embryonic (E10 to E18) and adult (6Wks) development (Fig. 2I and J). Additionally, the overall thickness of the retina was greater within the HAA compared to neighboring dorsal and ventral regions, with p-values of 7.4*10^−9^ and 1.5*10^−8^ respectively, throughout embryonic (E6 to E18) and adult (6Wks) development (Fig. 2K and L). To visualize this differential thickness within the HAA, retinal cross-sections through the HAA from early (E6) and late (E18) embryonic development were analyzed and heat maps of the total thickness of the retina along the radial axis were generated. Using these heat maps, we also observed that the thickest region of the cross-section, depicted in bright white color in the heatmap, overlapped with the developing HAA early in development and RFZ later during development. (Fig. 2M and N). These observations are consistent with the model of early specialization of the HAA in the developing retina.

### HCR method for visualization of chicken opsins

In humans, the very center of the fovea, the foveola, has no blue cones (Curcio et al. 1991), and has a highly variable ratio of red to green cones (Roorda and Williams 1999). We have little information regarding the distribution and ratio of the different cone subtypes in most organisms due to the lack of a method that allows identification and quantification of each cone subtype, especially in a single specimen and in specified areas, where it is most informative. However, in the posthatch day 15 (P15) juvenile chicken, Kram et al used colored oil droplets within cone inner segments, which correlate with opsin subtypes, to quantify different cone subtypes in the mid-peripheral retina (Kram et al. 2010). We wished to extend these characterizations to the HAA, and compare the cone distribution to other areas. To this end, we investigated whether HCR, which can be used to mark transcripts from multiple genes in a single specimen, could be used to mark each cone subtype in a whole mount, and allow for quantification.

**Figure 3.**
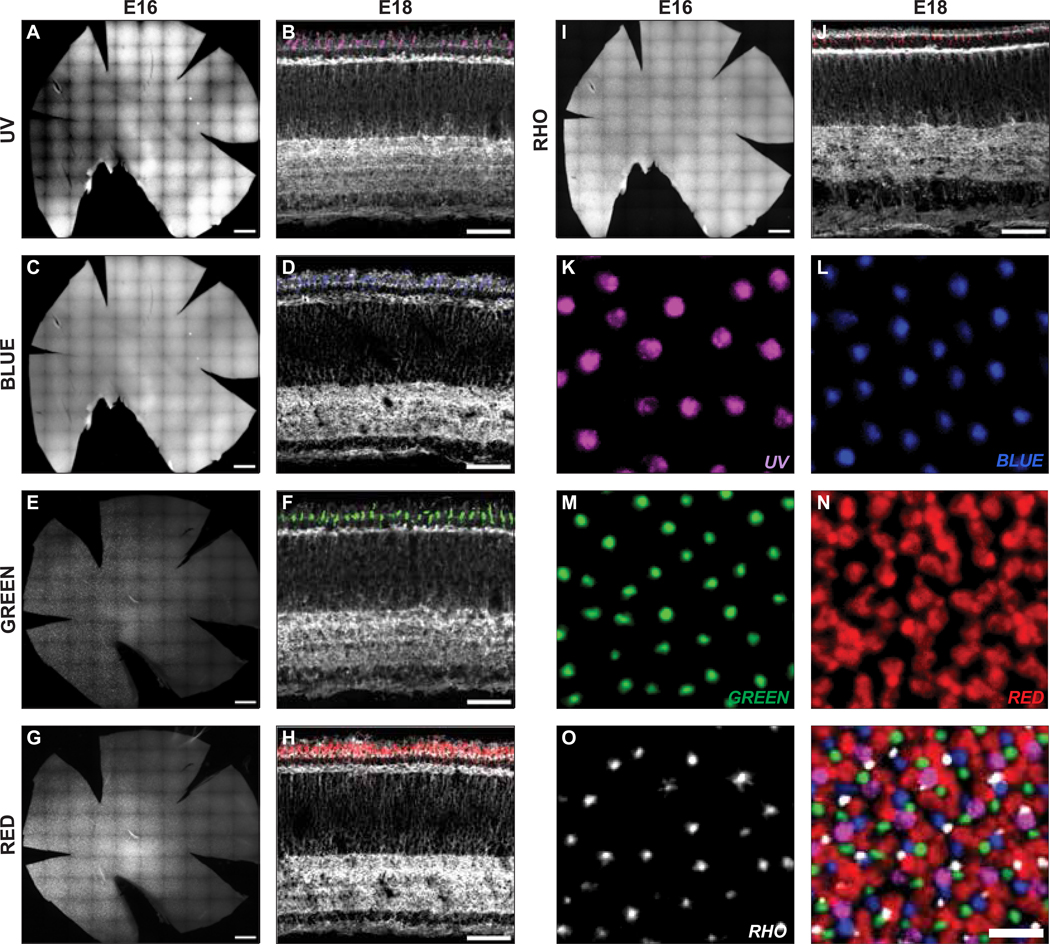
Expression patterns of embryonic opsins. **(A-J)** *In situ* HCR hybridization was conducted on retinal whole-mounts at E16 (A, C, E, G, I) and on cross-sections at E18 (B, D, F, H, J) using smFISH probes for opsins: UV (A, B), blue (C, D), green (E, F), red (G, H) and rhodopsin (I, J). **(K-O)** Multiplexed *in situ* HCR hybridization was performed on a single retinal whole-mount at E18 using the same set of probes for UV (K), blue (L), green (M), red (N) opsins and rhodopsin (O). **(P)** Images of each opsin from (K-O) were merged. Scale bars, 1000 μm (A, C, E, G, I); 50 μm (B, D, F, H, J); 10 μm (K, L, M, N, O, P).

The chicken retina possesses four subtypes of single cones and as well as a double cone type(s); single cones are referred to as UV, blue, green and red, based upon the spectral sensitivities of their opsin proteins, and double cones consist of pairs of closely apposed principal and accessory cones. While single cones are known to mediate tetrachromatic color vision, double cones are suggested to mediate detection of motion and/or magnetic field, but they have yet to be fully understood (Potier et al. 2017; Gunther et al. 2021; Seifert, Baden, and Osorio 2020; Wisely et al. 2017). Double cones have been shown to express red opsin (Araki et al. 1990; Hart 2001). Probes specific for UV, blue, green and red opsin transcripts along with *Rho* transcripts were hybridized to retinas harvested from E10 to E18. As previously reported using chromogenic detection methods, all opsin subtypes were detected on retinal whole-mounts from E16 onwards (Fig. 3A, C, E, G, I) (Bruhn and Cepko 1996). All of the opsins were expressed exclusively in the ONL, in keeping with the known specificity of opsin gene expression (Fig. 3B, D, F, H, J). The morphology of the red cones, as revealed by the HCR method, did not allow us to distinguish single red cones and double cones. Red opsin values are thus the sum of these cone subtypes due to the lack of distinct morphologies of double and single red cones revealed by the HCR signal.

To investigate if each cone expressed only one opsin subtype, multiplexed *in situ* HCR was conducted on a single retinal whole-mount using the probe sets for all types of opsin RNAs (Fig. 3K–O). When merged, one can see that the signals from each opsin subtype did not overlap with the signals of another opsin subtype (Fig. 3P). This finding demonstrates that each cone expresses a single opsin subtype. In addition, it shows the specificity of each probe set.

### Opsin expression in embryonic and adult chicken cones in the RFZ and nearby areas

**Figure 4.**
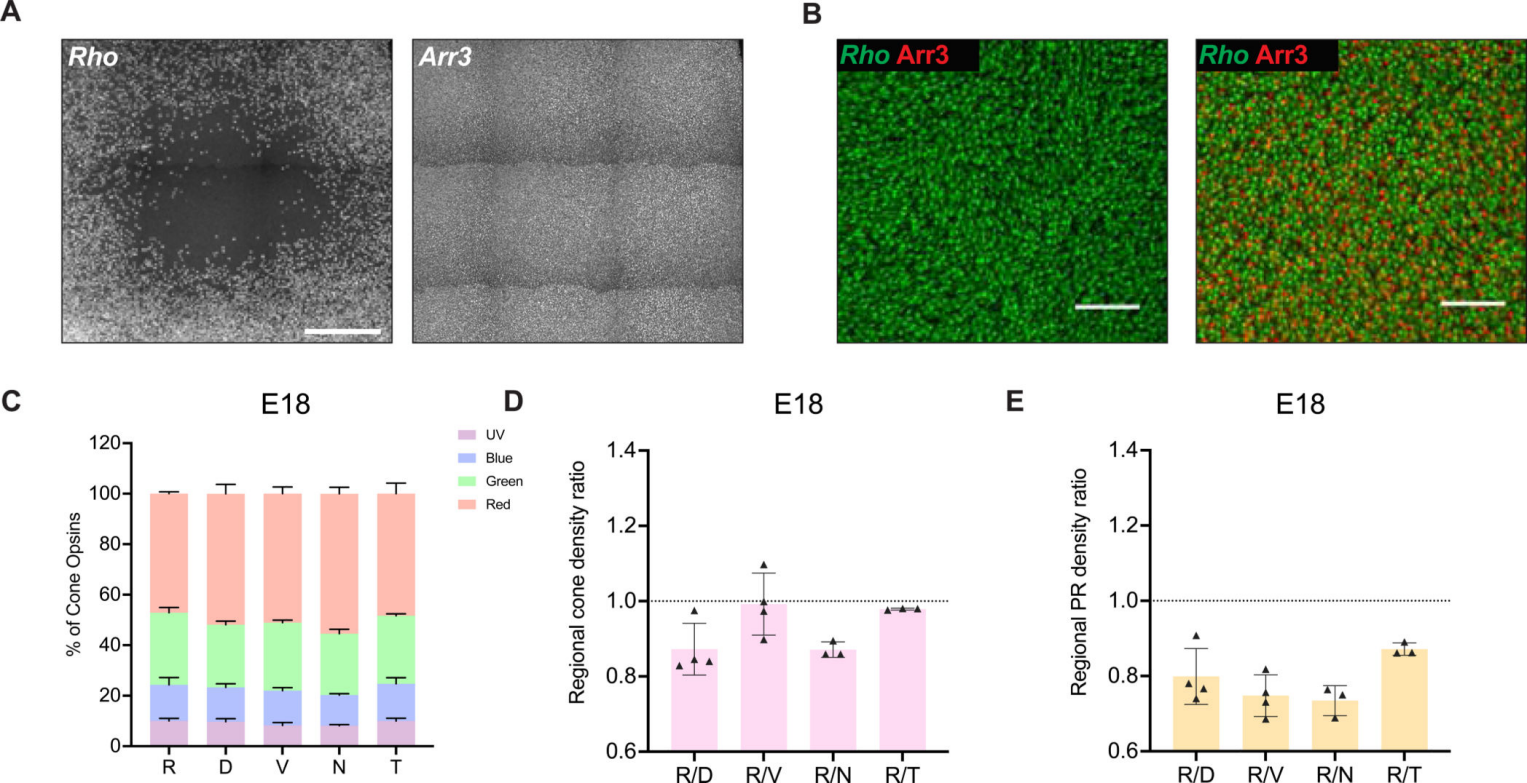
Compositions and densities of embryonic opsins. **(A)** *In situ* HCR hybridization was conducted on retinal whole-mounts at E18 using smFISH probes for *Rho* (left) and *Arr3* (right). Scale bar, 300 μm. **(B)** Images of *Rho* and *Arr3* from (A) were merged and shown within the RFZ (left) and outside of the RFZ (right). Scale bar, 50 μm. **(C-E)** *In situ* HCR hybridization was conducted on retinal whole-mounts at E18 using smFISH probes for individual cone subtypes and rods. The analyses of cone subtypes and rods within the RFZ and its neighboring regions are shown in C-E, with the composition of all cone photoreceptor subtypes (C), the regional total cone photoreceptor (sum of four subtypes) density ratio relative to the RFZ (D), and the regional photoreceptor (sum of rods and all cone subtypes) density ratio relative to the RFZ (E). R, RFZ; D, Dorsal; V, Ventral; N, Nasal; T, Temporal.

To compare the signals from the *Rho* HCR probes with our previous characterization, which used digoxigenin probes and a different ISH method, E18 retinal whole-mounts were hybridized with the HCR *Rho* probes. The location of the area devoid of HCR rhodopsin signals was consistent with the previous data (Fig. 4A). To ensure that the RFZ was not negative for rhodopsin HCR signals due to problems with hybridization in this area, the retina was probed for expression of arrestin3 (*Arr3*), a cone marker (Craft et al. 1994) (Fig. 4B). Dense *Arr3* signals were seen in this area, demonstrating the presence of HCR signals from this area, in the cone photoreceptors in the RFZ. To determine what subtypes of cones are present within the RFZ, the numbers of each opsin subtype were counted within the RFZ and neighboring regions, each 1 mm away from the RFZ. The RFZ exhibited expression of UV, blue, green and red opsins that account for 10.01±1.01%, 14.29±2.9%, 28.54±2.08% and 47.15±0.75%, respectively, of the total opsin count. These opsin patterns were very similar in the neighboring regions (Fig. 4C and Supplementary Fig. 3A).

In other animals with high acuity vision, the HAA has the highest cone density (Bringmann et al. 2018; Wassle and Boycott 1991). To test whether the chick retina also has the highest cone density in the RFZ, the ratio of the density of cones within the RFZ to the density of cones within its adjacent regions was calculated. The total cone density within each subregion was determined by summing the counts of all four cone subtypes per 10,000 μm^2^. Surprisingly, upon comparing the ratio of cone density within the RFZ to those of neighboring regions, the density of cones in the RFZ was found to be consistently the lowest across all E18 retinas (n=3) (Fig. 4D). Since rods were absent from the RFZ, the combined density of rod and cone photoreceptors was correspondingly lowest in the RFZ compared to its neighboring regions at the E18 timepoint (n=3) (Fig. 4E).

**Figure 5.**
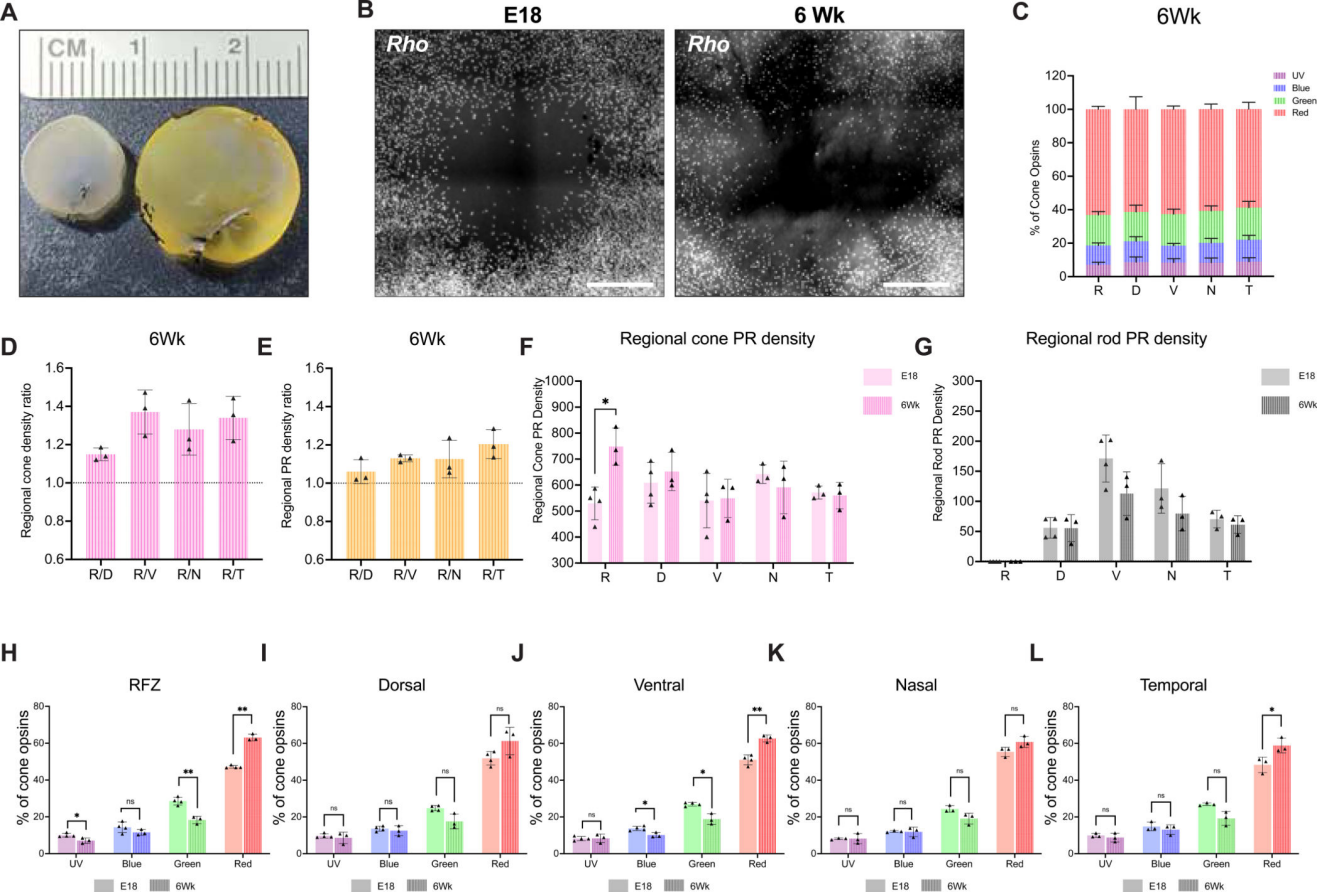
Compositions and densities of adult opsins. **(A)** Dissected retinas at E18 (left) and 6Wks (right). **(B)** The RFZ was located on retinal whole-mounts at E18 (left) and 6Wks (right) by *in situ* HCR hybridization with smFISH probes for *Rho*. Scale bar, 300 μm. **(C-L)** *In situ* HCR hybridization was conducted on retinal whole-mounts at 6Wks using smFISH probes for individual cone subtypes and rods. The analyses of cone subtypes and rods within the HAA and its neighboring regions are shown in C-F, with the composition of all cone photoreceptor subtypes (C), the regional total cone photoreceptor (sum of four subtypes) density ratio relative to the RFZ (D), the regional photoreceptor (sum of rods and all cone subtypes) density ratio relative to the RFZ (E), change in regional cone (F) and rod (G) photoreceptor density between E18 and 6Wks time points, and change in regional cone opsin composition between E18 and 6Wks time points (H-L). R, RFZ; D, Dorsal; V, Ventral; N, Nasal; T, Temporal; PR, Photoreceptor.

In humans, the fovea centralis takes a few years after birth to fully mature (Hendrickson 1992; Vajzovic et al. 2012). To investigate if additional time would allow chick retinal cones to reach a higher density in the HAA, 6-week-old (6Wks) chicken retinas were used for rod and cone opsin HCR (Fig. 5A). The RFZ was located within the adult retinal whole-mount based upon the absence of *Rho* HCR probe signal (Fig. 5B). Of note, the 6Wks adult retina is approximately 3 times larger in terms of the total volume of the eye globe compared to E18 retina (Fig. 5A). Interestingly, the proportional increase in size of the adult RFZ was much less than that of the increase in the entire retina (Fig. 5B). Taking into account that the genesis of photoreceptors is complete during early development in the chick retina (Belecky-Adams, Cook, and Adler 1996; Prada et al. 1991), it was possible that the overall cone density would gradually decrease posthatch as the volume of the retina increased. To determine if this was the case, the total number of each cone subtype was counted within the RFZ and its four adjacent regions in the 6Wks adult, with the expression of UV, blue, green, and red opsins, constituting 7.07±1.46%, 11.53±1.53%, 18.29±1.95% and 63.12±1.80% of the total opsin count, respectively. These opsin patterns were very similar in the adjacent regions, as depicted in Figure 5C and Supplementary Figure 3C. We performed several analyses to investigate regional changes in opsin composition between these two time points as described below.

First, to investigate if the cone density within the RFZ had changed by 6Wks relative to adjacent regions, we examined the ratio of the density of cones within the RFZ to the density of cones in the adjacent regions. Similar to the analysis of the E18 retinas, the total cone density within each subregion was determined by summing the counts of all four cone subtypes per 10,000 μm^2^. Interestingly, the density of cones in the RFZ was consistently the highest compared to the adjacent regions across all 6Wks retinas (n=3) (Fig. 5D). Moreover, even if rods were included in the count of photoreceptors, the density of photoreceptors was still the highest in the RFZ compared to its neighboring regions at 6Wks (n=3) (Fig. 5E). To investigate the fold change in density over time, rather than relative ratios at one time, we compared cone and rod photoreceptor densities between E18 and 6Wks within the RFZ and its neighboring regions (Fig. 5F and G). We observed a significant increase in the total cone density within the RFZ during posthatch development (Fig. 5F). Further analysis of the change in the composition of cone subtypes within the RFZ showed a significant increase in the number of cones that expressed red opsin between E18 and 6Wks (Fig. 5H). Moreover, cones that expressed UV and green opsin significantly decreased within the RFZ between these time points (Fig. 5H). Similar analysis of the neighboring regions revealed a significant increase in red opsin within the ventral and temporal regions and a significant decrease in green and blue opsin temporally (Fig. 5I–L). We reanalyzed previously published P15 opsin counts by Kram et al. (2010) and observed similar values for % opsins to those in E18 and 6Wks samples (Supplementary Fig. 3A–D). Furthermore, the values reported for the intermediate timepoint, P15, supported the trends observed between E18 and 6Wks (Supplementary Fig. 3A–D). This indicates a developmental trend in the chick retina, showing an increase in cones expressing red opsin and a decrease in cones that express other opsins after hatching.

### Pattern analyses of opsins

**Figure 6.**
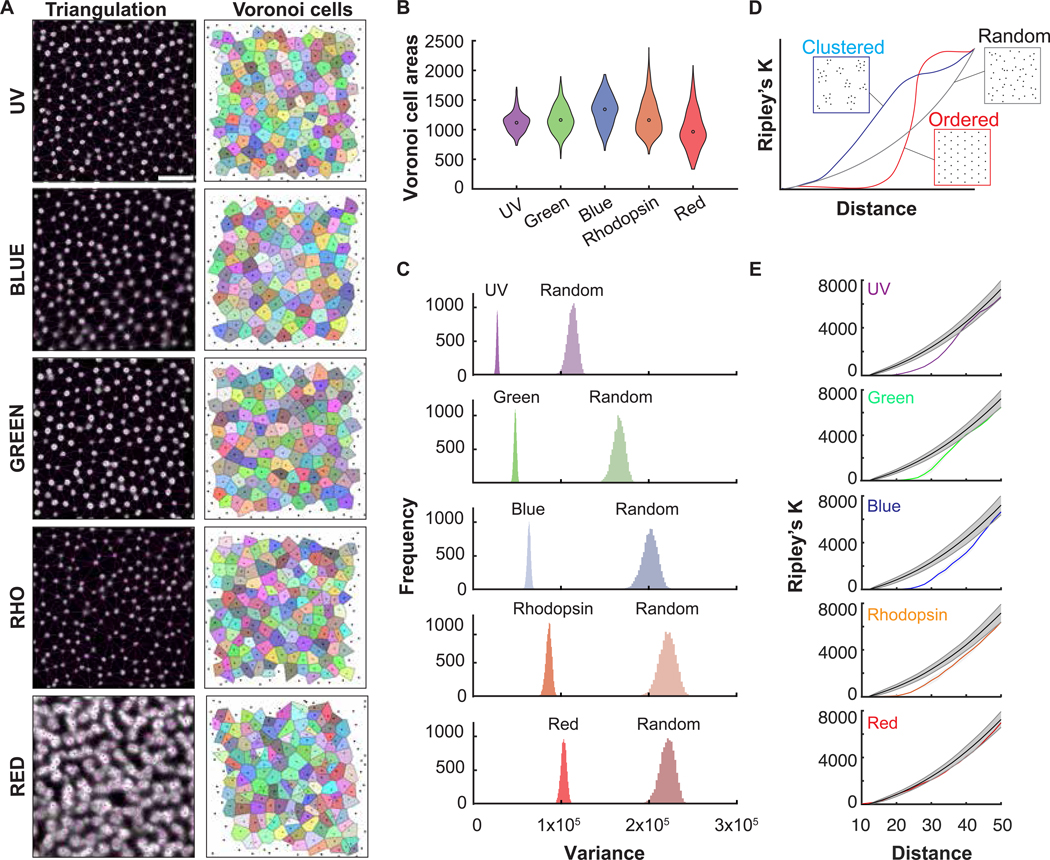
Pattern analyses of embryonic opsins. **(A)** Left column, representative HCR images for each opsin subtype (captured from temporal regions adjacent to the RFZ) overlaid with computationally extracted photoreceptor center points and Delaunay triangulation result in magenta. Right column, corresponding Voronoi tessellation plotted on a blank area of equal size to the original image. Photoreceptor center points are represented as black dots, and each Voronoi cell is labeled with a unique color. Scale bar, 25 μm **(B)** Areas of cells shown in (A, Right column) in μm^2^. **(C)** Frequency distributions generated from 10,000 samples of Voronoi cells. Samples were taken from opsin images (“UV”, “Green”, “Blue”, “Rhodopsin”, “Red”) and their corresponding “Random” simulated arrays (see Materials and Methods). **(D)** Hypothetical Ripley’s K curves for random (black), clustered (blue), and ordered (red) patterns as a function of distance. **(E)** Ripley’s K results for each opsin pattern, arranged from most ordered (top) to least ordered (bottom).

To further investigate the pattern of opsin expression, we employed two methods to analyze spatial order among photoreceptor subtypes in the E18 retina. For this purpose, HCR images of each cone subtype were analyzed from regions temporally adjacent to the RFZ (n=3 for each opsin). First, we semi-computationally extracted photoreceptor cell center points from HCR images. Then, Delaunay triangulation, which was used to generate a Voronoi tessellation for each image, was carried out (Fig. 6A). The variation in Voronoi cell areas between rods and cone subtypes provided an initial indication of pattern order, where a uniformly-sized group of Voronoi cells from an ordered cell tiling will have a lower variance. These single-image data indicated that cones expressing UV or green opsins had the most regularly patterned mosaics, while red opsin cones and rods were more randomly scattered (Fig. 6B). To further investigate this relationship, variances of thousands of random Voronoi cell samples extracted from each opsin image were analyzed. We then compared distributions of each cone subtype to each other subtype, and to the expected distributions if each of the subtypes were patterned randomly. All cone opsin subtypes, as well as rods, showed significantly lower variances in their cell area samples compared to the random cases (Fig. 6C). They also displayed a clear order from smallest to highest variance—UV, green, blue, and rhodopsin. As mentioned above, the signals from red opsin were more diffuse, requiring additional manual correction while detecting cell center points. In addition, the counts of cones that expressed red opsin were a combination of double cones and single cones.

As an additional measure of order, Ripley’s K function, a statistical point-pattern analysis method, was calculated from the positions of photoreceptor cells. Here, a given distance along the x-axis represents the radius of a circle drawn around each point, and the number of neighbors that fall within the circle is used to calculate K. Points that are arranged in complete spatial randomness (CSR) should show a linear increase in K with neighborhood size, while regularly arranged patterns have fewer nearby points than expected at short distances (Fig. 6D). In support of the Voronoi tessellation results, the curves for rods and all cone subtypes, except red, were more dispersed than random at the relevant distances for cell spacing. UV and green subtypes were the most ordered and rhodopsin the least (Figure 6E). These results are consistent with those of the previous analysis using colored oil droplets to characterize the cone mosaic in the mid-peripheral juvenile (P15) retina (Kram et al. 2010), that each opsin mosaic is made up of regularly arranged cell types.

### Apoptosis within the HAA during development

**Figure 7.**
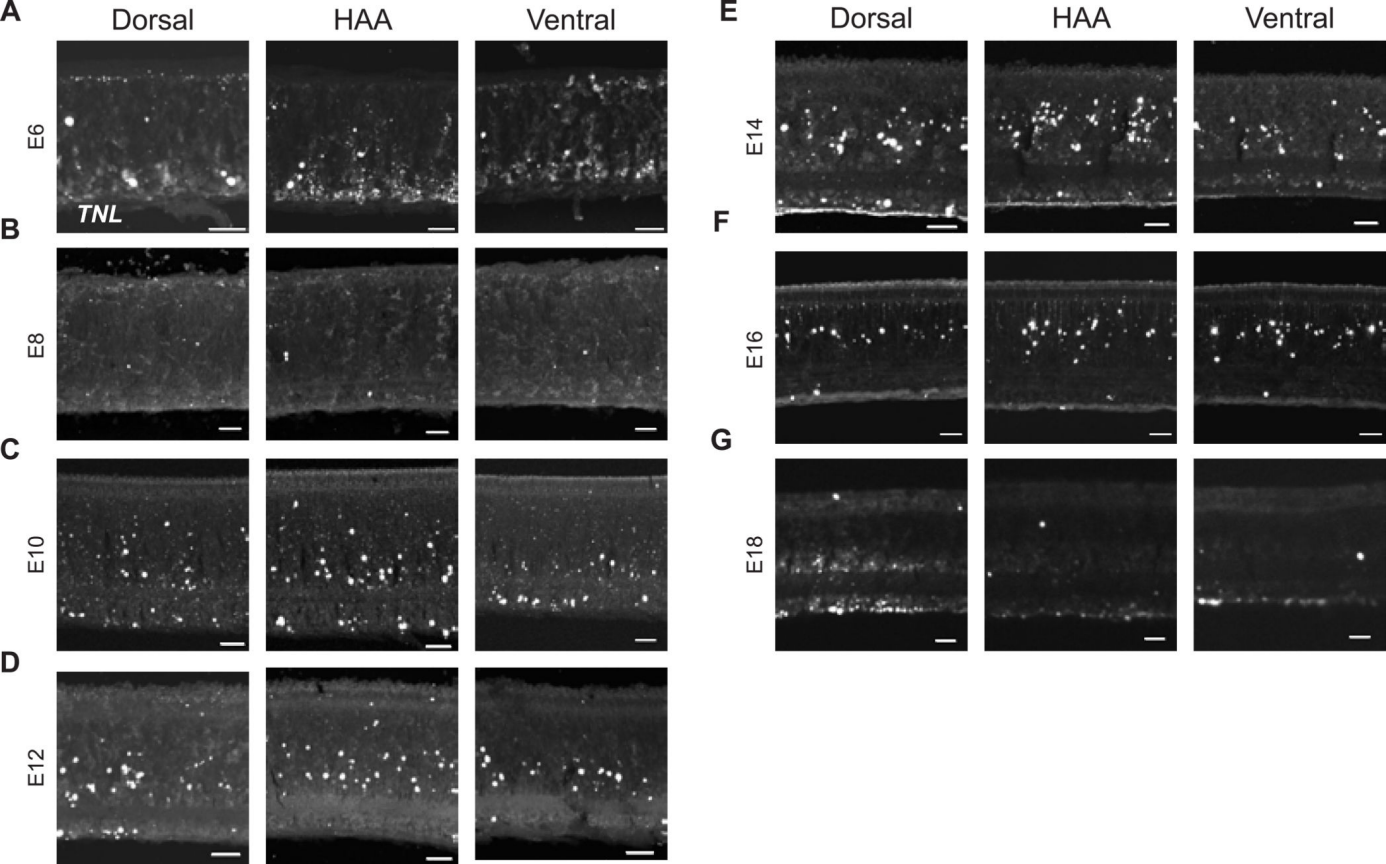
Apoptosis within the HAA during retinal development. **(A-G)** TUNEL staining was performed on retinal cross-sections of the HAA and neighboring dorsal and ventral regions (750μm away from HAA) using E6 to E18 retinas. The HAA was visualized using HCR *in situ* hybridization for *Fgf8* RNA on E6 to E14 retinas (A-E), and for *Rho* RNA on E16 and E18 retinas (F,G). Scale bar, 25 μm.

During chick retinal development, programmed cell death is known to contribute significantly to the final cell composition across retinal cell layers (Cook et al. 1998, Chavarria et al. 2007; Valenciano et al. 2009). We examined apoptosis within the HAA, with particular focus on whether there was additional apoptosis in the ONL which might account for the lack of rods in this area, and whether there was less death in the GCL, which might account for the higher density in this layer. TUNEL staining was performed on dorso-ventral cross-sections which included the HAA, from E6 to E18 retinas and the pattern of apoptotic cell death was compared between the HAA and adjacent regions (Fig. 7A–G). Almost no TUNEL^+^ cells were observed in the ONL within the HAA, or within the ONL of neighboring regions, throughout development, consistent with the observations reported in Cook et al. (1998). Moreover, a sparse pattern of TUNEL^+^ cells was observed in the GCL within the HAA, as well as within the neighboring regions, throughout development. A previous study of cell death patterns in the chick GCL reported less cell death in the central retina compared to the peripheral retina (Straznicky and Chehade 1987). No significant difference was observed here between the amount of apoptosis in the GCL within the HAA and its neighboring region, likely due to the fact that all of the regions that we analyzed were located very close to each other in the central retina (Supplementary Fig. 2B).

## DISCUSSION

We set out to characterize the morphological and cellular characteristics of the developing HAA in a tractable model organism. We first needed to find a marker that would allow its definition throughout development and into the adult stages. In addition, a method, HCR, for detection of multiple genes within retinal whole mounts and sections was explored as a way to learn of photoreceptor patterns over time at high resolution. This method, along with more classical ones, allowed for a description of changes in cellular densities and cell subtypes within the developing and mature HAA and its surrounding areas.

### High *Fgf8* marks the developing HAA

*Fgf* signaling has been shown to be critical for retinal patterning and fate determination across multiple species, including mice, zebrafish, and chicken (Makrides et al. 2022; Martinez-Morales et al. 2005; da Silva and Cepko 2017; Nakayama et al. 2008; Picker and Brand 2005). Our group recently reported that *Fgf8* is expressed in a discrete spot consistent with the developing chick HAA, starting early in development before most neurons are born. In the present study, we extended this analysis, demonstrating that it marks this area throughout development. Importantly, the use of HCR to show expression of more than one gene at high resolution in the same cells showed that *Fgf8* indeed marks the RFZ. This discrete expression pattern and functional role of *Fgf8* in formation of this area suggest a model of distinct types of RPCs within the early anlagen that produce the specific cohort of cell types within the HAA. Consistent with this model are data from our previous characterization of RNAs expressed in *Fgf8*^*+*^ and *Fgf8*^−^ cells from chick E6, which exhibited multiple differentially expressed genes (Amamoto et al. 2019). Further definition of the properties of *Fgf8*^*+*^ cells will be required to fully investigate this model.

The pattern of *Fgf8* expression shows an interesting distinction along the dorsoventral axis. While *Fgf8* expression is diffuse dorsally, it has a very sharp border ventrally during the early stages of development. A transcription factor, *cVax*, is expressed in the ventral retina and has a sharp dorsal border. It is expressed very early, in the optic vesicle, and has been shown to determine ventral polarity in the retina (Mui et al. 2005). We showed that lack of RA is important for *Fgf8* expression as well as patterning of the HAA (da Silva and Cepko 2017), and *cVax* has been shown to be upstream of RA signaling in the chick retina (Golz et al. 2008). Additionally, we previously misexpressed *cVax* throughout the retina and found that the RFZ was absent (Schulte et al. 1999). These findings position c*Vax* to be a crucial part of the patterning machinery that establishes the ventral border for *Fgf8* and places the HAA along the equator.

### Specialization of the GCL within the HAA during embryonic development

One of the earliest signs of a developing HAA is a thickening within the central region, observed in multiple organisms (Kelling et al. 1989; Mann 1964; Rasys et al. 2021; Reichenbach et al. 1991). We measured the thickness of individual retinal layers, and compared these values between the HAA and its neighboring regions. We found that the GCL was consistently thicker within the prospective HAA relative to the neighboring regions throughout embryonic development (Fig. 2I and J). The functional significance of a thicker GCL, with presumably more RGCs, is that it allows for reduced convergence of cones onto RGCs, thereby contributing to higher acuity (Kolb et al. 1995). The observation of a thicker GCL, or greater retinal thickness, in the developing HAA across species is consistent with genesis of more neurons in this region. Importantly, the peak of chick GCL birthdays was found to be in the central retina at E5-E6 (Prada et al. 1991; Belecky-Adams, Cook, and Adler 1996). As we observed a thicker GCL in the HAA, relative to nearby regions, at E6, there is likely increased genesis of GCL cells in this area. This is consistent with the above model of a distinct pool of RPCs in this area, as opposed to a central to peripheral gradient of genesis of retinal neurons, i.e. the adjacent regions that we measured were right next to the HAA and thus would not be expected to be very different in terms of central-peripheral birthday kinetics. After E12, the GCL thickness throughout the HAA and adjacent regions began to gradually increase, although no additional neurons should be generated (Prada et al. 1991; Belecky-Adams, Cook, and Adler 1996), perhaps due to an increase in the soma size. Alternatively, or additionally, the GCL may expand to accommodate the increasing dendritic projections from this layer to the inner plexiform layer (IPL), which increases ~2.5x in thickness from E16 to 6Wks (Supplementary Fig. 2A). Overall, the higher GCL thickness at the HAA was established early, during the period of genesis, and was maintained for the remainder of embryonic development.

Differential cell death also could be playing a role in establishing the higher density of cells within the GCL of the HAA. We observed little cell death in the GCL compared to the INL and ONL within the HAA and its neighboring regions throughout development. These observations are consistent with those of a previous study wherein less cell death was observed in the central GCL of the chick (Straznicky and Chehade 1987). Lack of an early marker of the HAA precluded an analysis of cell death specifically within the HAA in this previous study. Collectively, these observations suggest that a distinctive pattern of neurogenesis, as well as a modest contribution from less cell death, establish the specialized GCL within the HAA during early stages of chick retinal development.

Approximately 70% of RGCs in the primate central retina are of the “midget” subtype, characterized by their relatively smaller cell bodies and dendritic trees compared to other RGC subtypes, with their smaller size enabling the central retina to accommodate more RGCs compared to the periphery (Dacey 1993; Polyak 1941; Marshak 2009). The midget system exhibits differential circuitry, having a one-to-three ratio between cones and RGCs, which is critical for high acuity vision (Dacey 1993; Kolb, Fernandez, and Nelson 1995; Remington 2012). Although very little is known about the RGC circuitry in the chicken HAA, certain RGC subtypes have been observed which are smaller in size and are thought to be connected with a smaller number of bipolar cells (Baden and Osorio 2019). ‘Midget-like’ bipolar cell circuitry also has been reported in the chicken retina, with a single bipolar cell connected to a single cone (Quesada, Prada, and Genis-Galvez 1988). These observations also are consistent with the model of a specialized pool of RPCs in the developing HAA, which leads to the genesis of distinct neuronal subtypes in this area.

### The cone mosaic in the HAA

An analysis of cone subtype frequencies and arrangement had not been reported for the HAA, though our previous study, which was low resolution due to the use of digoxigenin-labelled in situ hybridization probes, showed that all cone subtypes were present in the HAA. Modern FISH methods provide an opportunity to quantify the individual cone subtypes within the HAA and other regions. These methods open up the possibility of quantitative analysis of cone subtypes across species, which has not been possible in most species due to lack of specific reagents. Our data show the power of this method, both on whole mounts, where one can most easily appreciate patterns, and in sections. Such methods can leverage the voluminous scRNA-seq data, for any cell type in a sequenced organism. Application of the cone probes in our current study provided several noteworthy observations.

Our observations of cone density over time can be integrated with the findings of several previous studies, and suggest some additional ideas. First, we found that the abundance of cone subtypes was in the order of red, green, blue and UV cones within and outside of the HAA in both embryonic and adult retina, with red cones being the most abundant, consistent with a previous report (Kram et al. 2010). Moreover, as our group has shown before (Bruhn and Cepko 1996), rods were more abundant ventrally than dorsally despite varying proportions of rods within the neighboring regions of the HAA, also consistent with the data from Kram et al. (2010). However, we did not observe a dorsal-ventral gradient in the UV and blue opsin counts, as was reported by Kram et al. (2010) in their study of the mid-peripheral retina, likely due to the fact that all of the regions that we analyzed were located very close to each other in the central retina (<2% of the retinal surface area at E18 and <1% of the retina surface area at 6Wk).

Second, investigation of photoreceptor patterns using Voronoi tessellation analysis showed that cones display clear differences in their regularity. UV cones are the most ordered photoreceptor subtype, followed by green, blue, and red subtypes, then rods. Spatial pattern analysis using Ripley’s K function revealed second-order properties of point distributions, such as the degree of dispersal or clustering. By this method, all photoreceptor subtypes are “overdispersed”, or significantly more ordered than expected for randomly arranged cells. Notably, red is the most disordered cone subtype in our analysis, possibly because we were unable to parse single from double cone forms. Kram et al. (2010) found that double cones were more regularly spaced than single, making it possible that single and double red cones display distinct mosaics that collapse into a disordered array when viewed in full. Given the importance of even cell distribution across the retina for parallel information processing, the embryonic development of highly regular cone patterns in the chick is unsurprising (Wassle 2004). Though rods form even rows in zebrafish (Fadool 2003), and dispersed rod patterns have been reported in mice (Reese and Keeley 2015), their pattern relative to cones has not been characterized in most species

Third, counter to our expectations based upon the features of the early HAA GCL, the late embryonic HAA did not exhibit a higher density of cones compared to its neighbors. However, the adult HAA was found to have a higher density of cones relative to those of the adjacent regions. Interestingly we found that the HAA maintained a similar size between late embryonic and posthatch timepoints, while the eye globe increased ~3 times in volume. It is possible that the cones in this region have homotypic adhesion that, during the posthatch period, leads to the higher cone density. In this scenario, cones from the immediately adjacent regions would be recruited into the RFZ, to maintain the size of this region. Rods might be excluded from this process, or might die during the process. Differential expansion also might contribute to the increase in density of cones in the HAA relative to its adjacent regions. Such differential expansion has been suggested to play a role in establishing distinctive cellular densities between the HAA and surrounding regions in other species (Kelling et al. 1989; Rasys et al. 2021). Multiple mechanisms can account for this. There may be differential tethering of the HAA to the vitreous (Brewton and Mayne 1992; Coulombre, Steinberg, and Coulombre 1963; Wright and Mayne 1988), which may extend into the adult period, and be at least partially responsible for pathologic posterior vitreous detachment seen in humans (Tsui et al. 2012; Johnson 2005; Kishi et al. 1986; Liu et al. 2014). The RPE may also play a role, as in the human retina, in the foveal area, there is an increase of the RPE cell density, and distinctive cell shapes, perhaps due to a centripetal shifting of cells towards the macular area (Robb 1985; Ortolan et al. 2022). There may also be asymmetric elongation due to a differential elasticity of the HAA relative to its adjacent regions (Rasys et al. 2021; Kelling et al. 1989; Springer 1999; Springer and Hendrickson 2004). While the mechanisms proposed above could address how the HAA maintains a similar size between late development and the adult stages, we did not observe a reduction in photoreceptor density between E18 and 6Wks in the adjacent regions. We speculate that this is since these adjacent regions were extremely close to the HAA and hence would not reflect expansion of the more peripheral retina (<2% of the retinal surface area at E18 and <1% of the retina surface area at 6Wks).

Fourth, in keeping with the increase in relative density of cones within the RFZ relative to surrounding regions, we observed a significant increase in cones that expressed red opsin at 6Wks relative to E18, in the RFZ. There was also a slight increase in red opsin cones in the ventral and temporal adjacent regions. There was a decrease in green, blue, and UV opsins in the RFZ, though not enough to account for the increase in red opsin. Given retinal birthdating data from the chicken demonstrating that all photoreceptors are born by mid-embryonic times (Belecky-Adams, Cook, and Adler 1996 1996; Prada et al. 1991), it is unlikely that the increase in red opsin cones was due to cone genesis. One possibility is that there is a late onset of red opsin expression in some subtypes of cones. While at least three subtypes of double cones with distinct morphologies have been identified in the adult chicken retina (Wai et al. 2006), the kinetics of red opsin expression in each subtype has not been addressed, and cone differentiation continues until P15, (Wai and Yew 2002; Wai et al. 2006; Lopez et al. 2005). Additionally, the movement of red cones towards the foveal area in the human retina during foveal maturation has been suggested (Diaz-Araya and Provis 1992; Hendrickson 1994) and could occur in the chicken. Lastly, a cone subtype might switch to a different opsin subtype (red) during posthatch development, as opsin switching can occur in some species (Cheng and Flamarique 2007; Glaschke et al. 2011), though the decrease in other opsins does not equal the increase in red in the RFZ. Birthdating studies and investigations into the timing of opsin onset relative to birthdate and their position in the retina could provide valuable insights into these potential mechanisms.

Lastly, the relative ratios of different cone subtypes in the embryonic and adult HAA were highly similar to those of neighboring regions (Fig. 4C and 5C; Supplementary Fig. 3A–D). These observations imply that cones are generated both within and outside of the HAA independently of rod formation. Cone mosaic formation also has been studied in the retina of goldfish, where rods are randomly incorporated into the cone mosaic throughout the juvenile and adult periods (Johns 1982). However, while cones are born a few days prior to rods in the goldfish, the birthdates of rods vs cones have not been determined in the chick (Prada et al. 1991; Belecky-Adams, Cook, and Adler 1996). Regardless of whether rods contribute to the establishment of the cone mosaic, another important question is how the HAA becomes devoid of rods. While almost no apoptotic events were observed in the developing chick ONL (Fig. 7), it is worth noting that other types of cell death were not assayed and might potentially occur (Cook et al. 1998). It is possible that rods migrate out of the RFZ during and/or after their genesis, but no increase in rod density in the immediate vicinity of the HAA was observed (Fig. 3I). Alternatively, we favor the model suggested above of a distinct pool of RPCs that create the HAA, with HAA RPCs being incapable of producing rods. As the fundamental mechanisms by which rods vs cones are determined is unclear, we cannot address this at the moment. However, the system under study here may reveal such mechanisms, as well as provide a link to the overarching patterning mechanisms for this important area.

## CONCLUSION

Our observations provide foundational information regarding the development of the HAA in the chick retina. We were able to focus on the patterns of genesis and cell death, as well as changes in retinal layers, in the developing HAA by demonstrating that this area is marked by *Fgf8* expression throughout development. The results suggest that there are distinct *Fgf8*+ RPCs in the HAA which generate more RGCs, and fail to produce rods. The final pattern of cellular layers is consistent with differential (less) expansion of the HAA relative to more peripheral areas. These findings suggest a strategy used by the retina to create this special area, and set the stage for studies of developmental mechanisms in the chick where perturbations at early stages in development are readily carried out.

## MATERIALS AND METHODS

### Animals

Embryonic retinal tissues were obtained from fertilized White Leghorn eggs (Charles River). Fertilized eggs were incubated at 38°C with humidity around 40% and embryos were staged according to Hamburger and Hamilton staging (Hamburger and Hamilton 1951). Adult retinal samples were collected from fresh 6-week-old adult rooster heads obtained from AVS Bio in 1X PBS at 4°C.

### Tissue preparation

Right retinas were dissected in 1X PBS. To remove the RPE, the retinas were incubated in a 0.1 mg/mL Dispase I (Sigma-Aldrich, D4818–2MG) solution (prepared by dissolving 2 mg in 20 mL distilled water) for 10 minutes at room temperature (RT). Following the removal of the RPE, the retinal samples were fixed for 30 minutes in 4% paraformaldehyde (v/v). After fixation, retinas were washed 3 × 5 minutes in 1X PBS at RT before proceeding with HCR RNA-FISH as described below. To obtain retinal cross-sections through the HAA, dorsal-ventral rectangles were cut out from imaged flat mounted tissue after HCR RNA-FISH for *Fgf8* or *Rho*. The retinal pieces were then incubated in 30% sucrose in PBS (w/v) overnight at 4°C, and transferred to a 1:1 solution (v/v) of 30% sucrose in PBS:OCT for 3 hours. The samples were then frozen in cryomolds and stored at −80°C. For each dorsal-HAA-ventral sample, 30μm retina cryosections were collected and placed onto a cold superfrost plus microscope slide (Fisherbrand) within the cryostat, and stored at −80°C.

### Flat Mount HCR *in situ* hybridization

HCR in situ hybridization was performed as described previously (Choi et al, 2018) with some modifications. Probes were manufactured by Molecular Instruments based on the sequences provided as follows: *Fgf8* (NM_001012767.1), *Opn1sw* (NM_205438.1), *Opn2sw* (NM_205517.2), *Opn1msw* (NM_205490.1), *Opn1lw* (NM_205440.2), *Rho* (NM_001030606.1), *Arr3* (NM_001097533.2). Retinas were fixed with 4% paraformaldehyde (v/v) overnight at 4°C, washed in PBS twice at RT for 5 minutes each, fixed in 70% ethanol/PBS for 2~4 hours at RT, incubated in washing buffer at RT for 10 minutes, incubated in hybridization buffer at 37°C for 30 minutes, and hybridized with probes overnight at 37°C. Next day, retinas were washed in washing buffer twice at RT for 30 minutes each, washed in 5X SSCT with 0.1% Tween-20 twice at RT for 20 minutes each, and incubated in amplification buffer at RT for 10 minutes. To detect the probe, amplifiers were heated at 95°C for 90 seconds, incubated at RT for 30 minutes in the dark, mixed with the retinas, and incubated overnight at RT. Next day, retinas were washed in 5X SSCT with 0.1% Tween-20 twice at RT for 30 minutes each. Subsequently, retinas were mounted and imaged. For multiplexing, after the first round of imaging, coverslips were removed in PBS, retinas were demounted from the slide and treated with DNase (Roche, 4716728001) (1:50 dilution, for example, mix 30 μL of DNase with 150 μL of 10X buffer and 1,320 ul of distilled water) for 1 hour at 37°C. Subsequently, the existing DNase was removed, and retinas were incubated in new DNase O/N at 37°C. The next day, retinas were washed twice with 2X SSC containing 65% formamide (Millipore Sigma, F9037)(For example, mix 1 mL of 20X SSC with 6.5 mL of formamide and 2.5 mL of distilled water) for 30 minutes each at 37°C. Then, retinas were washed with 5X SSCT for 30 minutes at RT, followed by the second round of hybridization as described above.

### Immunofluorescence on cryosections

Following HCR *in situ* hybridization for *Fgf8* and *Rho*, cryosections were blocked in blocking buffer (4% donkey serum, 0.3% BSA, 0.3% Triton X-100 and PBS) for 2 hours at RT. Cryosections were incubated with a GS primary antibody (ThermoFisher, GT1055) diluted in blocking buffer overnight at 4°C. Next day, cryosections were washed in PBS three times at RT for 5 minutes each, incubated with a secondary antibody (Alexa Fluor 488, ThermoFischer, A-11001) diluted in blocking buffer for 1 hour at RT, washed in PBS twice at RT for 5 minutes each, stained with NucBlue (ThermoFisher, R37606) for 20 minutes at RT, washed in PBS twice at RT for 5 minutes. Subsequently, cryosections were mounted and imaged.

### Analysis of the retinal thickness

The images of *Fgf8* and *Rho* on cryosectioned retinas were analyzed in ImageJ. The HAA was identified on the dorso-ventral retinal sections as the region with high *Fgf8* expression in E6-E14 retinas and as the RFZ in E16, E18 and 6Wks retinas. The retinal thickness was measured within the HAA and 750μm away from the HAA towards dorsal and ventral retina, respectively. Relative intra-retinal GCL and total retinal thickness ratios corresponding to the HAA were plotted using the GraphPad software. Three retinas were used for these analyses. Retinal and layer thickness were measured in three subregions in the HAA as well as the dorsal and ventral regions (Refer to Supplementary Table 1). These values were averaged to obtain measurements specific to each region within a retina. Intra-retinal trends were observed by comparing thickness measurements within different regions of the same retina. While a high degree of variation in absolute values of the measurements was observed between animals, the intra-retinal trends noted here were observed consistently. The p-values for respective HAA/dorsal thickness, HAA/ventral thickness ratios being >1 were calculated using the binomial test. The retinal thickness heatmaps were generated using ImageJ. These heatmaps were constructed from composite images of E8 and E18 dorso-ventral retinal sections through the HAA, which were stained with *Fgf8*, *Rho* and NucBlue. The images were processed using the Kuwahara filter and Gaussian Blur operation to reduce adaptive noise and produce a smoother appearance. Following that, thresholding was applied to facilitate identification of radial boundaries and a distance map was generated using a skeletonization process. This distance map was utilized to determine the coloration of the heatmap, highlighting variations in thickness across the section. Areas depicted in red or brighter colors correspond to regions with greatest distance from center to the edge, while those in blue or darker shades represent smaller distance from the center to the edge along the radial axis.

### Analysis of the opsin compositions

The images of all opsins on flat-mounted retinas were analyzed in ImageJ. For each animal, opsin subtypes were counted within five distinct regions - the RFZ and the neighboring dorsal, ventral, nasal, temporal regions - each situated 1 mm away from the RFZ in the respective directions. For each individual region, opsin subtypes were counted in three sub-regions, each spanning an area of approximately 9,000 to 16,000 μm^2^. To account for variations in the size of the sub-region that was assayed, all opsin counts from the sub-regions were normalized to the measured area. Opsin density was then calculated for all subtypes in each subregion as opsin count per 10,000 μm^2^. The resulting opsin densities from the three subregions were averaged to obtain representative density counts for each opsin subtype in an individual region. This analysis was repeated to obtain opsin subtype densities from five distinct regions - the RFZ and the neighboring dorsal, ventral, nasal, temporal regions. Regional photoreceptor and cone densities were calculated from E18 (n=3) and 6Wks (n=3) retinas and plotted using the GraphPad software (Refer to Supplementary Table 2). Data from E18 animal#2 was excluded from downstream analysis as it exhibited outlier opsin counts (Supplementary Fig. 3E). The relative densities across regions were calculated by obtaining the ratio of photoreceptor and cone densities within the RFZ relative to the surrounding four regions for each animal. The change in regional rod and cone density between E18 and 6Wks timepoints was plotted using average regional counts and the p-values were calculated using two-tailed Student’s T-test, assuming unequal variance. The average density of each cone opsin type was used to calculate regional opsin compositions. The change in regional opsin composition between E18 and 6Wks timepoints was plotted and the p-values were calculated using two-tailed Student’s T-test, assuming unequal variance. Previously published opsin density and composition data from P15 retina (Kram et al. 2010) was reanalyzed by summing the red single and double opsin counts and further comparing with our E18 and 6Wks data points (Refer to Supplementary Table 2).

### Spatial order analysis

HCR images of each opsin type from regions temporally adjacent to the RFZ were analyzed from E18 retinas (n=3). Two spatial order analyses were performed to determine the relative degree of patterning among subtypes in the E18 retina. Using a custom MATLAB image analysis pipeline, cell centroids were first extracted and stored for each photoreceptor subtype from binarized multiplex HCR images. Cell identification in MATLAB involved an initial automated step using the *centroid* function, followed by manual correction to subtract erroneous calls and add missing cell center points. Images were cropped such that the number of cells per image was approximately equal. *delaunay* and *voronoi* MATLAB functions were used to extract Delaunay triangle edge lengths and Voronoi cell areas. To generate variance distributions, 10,000 random samples of size ¾N were taken for each set of N Voronoi cells. Random distributions for comparison were simulated for each cell subtype by first generating an array of points in a blank field with the same dimensions as each cropped HCR image. Given the well-described “exclusion zones” observed for different photoreceptor cell subtypes, the minimum distance between neighboring random points for each case was designated as the smallest distance between two cells of the same subtype (Reese and Galli-Resta 2002).

Ripley’s K was calculated as:

(1) $$K(d)=\frac{R}{n^{2}}\Sigma \underset{i\neq j}{\Sigma }\frac{I_{d}(d_{ij})}{w_{ij}}$$

where R is the area of interest, n is the number of points within a circle of radius d drawn around each point, and $d_{ij}$ is the distance between the center point and each neighboring point. If $d_{ij}<d$, $I_{d}(d_{ij})$ equals 1 (Okabe and Yamada 2001). The CSR curve and confidence intervals were determined from 100 iterations of randomly generated point arrays for each opsin subtype.

### TUNEL assay

The TUNEL assay was performed using the Click-iT^™^ Plus TUNEL Assay for in situ Apoptosis Detection, Alexa Fluor^™^ 488 dye (Invitrogen, Carlsbad, CA, USA) as per the manufacturer’s protocol. Retinal cryosections were rehydrated with 1X PBS, covered with terminal deoxynucleotidyl transferase (TdT) reaction buffer, and incubated at 37 °C for 10 min. Following that, the sections were covered with TdT reaction mixture and incubated at 37 °C for 1 h. Sections were then rinsed with distilled water, incubated in 3% (w/v) bovine serum albumin (BSA) with 0.1% Triton-X 100, and rinsed with PBS. The sections were then covered with freshly made Click-iT^™^ Plus TUNEL reaction cocktail and incubated at 37 °C for 30 min. After being incubated in 3% BSA and rinsed with PBS, sections were stained with NucBlue (ThermoFisher, R37606) for 20 minutes at RT, washed in PBS twice at RT for 5 minutes. Subsequently, cryosections were mounted and imaged. Images were analyzed using ImageJ to count TUNEL+ apoptotic cells. The apoptotic index was plotted using the GraphPad software. Three retinas were used for these analyses. While a high degree of variation in absolute values of the measurements was observed between animals, the intra-retinal trends noted here were observed consistently.

### Microscopy

Samples were imaged with a Yokogawa CSU-W1 single disk (50μm pinhole size) spinning disk confocal unit attached to a fully motorized Nikon Ti2 inverted microscope equipped with a Nikon motorized stage and an Andor Zyla 4.2 plus sCMOS monochrome camera using a Plan Apo λ 20x/0.8 DIC I and Plan Fluor 40x/1.3 Oil DIC H/N2 objective lens. Nikon Elements AR 5.02 acquisition software was used to acquire the data. Z-stacks were acquired using Piezo Z-device, with the shutter closed during axial movement. Data were saved as ND2 files.

## Supplementary Material

MMC1 Supplementary Table 1. Retinal layer thickness during chicken development.
The table presents raw counts of retinal layers within the HAA and its neighboring regions from embryonic (E6 to E18) and adult timepoints (6Wks), along with examples of analyses performed and p-value calculations. HAA, High Acuity Area.

MMC2 Supplementary Table 2. Compositions and relative densities of opsins in E18 and 6Wks retinas.
The table presents raw and analyzed photoreceptor counts from within the RFZ and its neighboring regions, along with examples of analyses performed and p-value calculations. RFZ; Rod Free Zone.

MMC3 Supplementary Figure 1. Morphological development of the chicken HAA.
**(A-P)** Retinal cross-sections of the HAA, neighboring dorsal and ventral regions from E6 to 6Wks. The HAA was visualized in E6 to E14 retinas using HCR *in situ* hybridization for *Fgf8* RNA (A, C, E, G, I), and for *Rho* RNA on E16, E18 and 6Wks retinas (K, M, O). Dorsal-HAA-Ventral sections were further stained with NucBlue to visualize retinal cell layers (B, D, F, H, J, L, N, P). Scale bar, 25 μm.
Supplementary Figure 2. Retinal thickness and apoptotic cell death during chicken development.
**(A)** Line graph comparing IPL thickness E16 to 6Wks within the HAA and neighboring dorsal and ventral regions in retinal cross-sections. Three retinas per timepoint were quantified. **(B)** Bar graph of number of TUNEL+ cells/10,000 μm^2^ in the GCL within the HAA and neighboring dorsal and ventral regions during chick retinal development. Three retinas per timepoint were quantified. IPL, Inner plexiform layer; GCL, Ganglion cell layer.
Supplementary Figure 3. Composition and densities of embryonic and adult opsins
**(A, C)** *In situ* HCR hybridization was conducted on retinal whole-mounts at E18 and 6Wks using probes for individual cone subtypes and rods. The regional composition of all cone photoreceptor subtypes is shown for E18 (A) and 6Wks (C) time points. **(B, D)** Cone opsin counts from P15 chicken retinas previously published (Kram et al. 2010) were reanalyzed to align with cone counting methods used in the current study. The regional composition of all cone photoreceptor subtypes is shown. **(E)** Regional cone photoreceptor density counts from E18 data, with and without Animal#2 which showed outlier values. R, RFZ; D, Dorsal; V, Ventral; N, Nasal; T, Temporal; DN, Dorsal-Nasal; DT, Dorsal-Temporal; VN, Ventral-Nasal; NT, Ventral-Temporal.

## Data Availability

Raw counts of retinal layers at HAA and its neighboring regions from embryonic (E6 to E18) and adult development (6Wks), along with examples of analyses performed and p-value calculations can be found in Supplementary Table 1. Raw and analyzed photoreceptor counts from the HAA and its neighboring regions, along with examples of analyses performed and p-value calculations are available in Supplementary Table 2. Source code for opsin pattern analysis can be accessed on Github (https://github.com/hasreetkgill/opsinanalysis).

## Highlights

- Fgf8 provides a molecular marker for the developing chick high acuity area (HAA).
- The retinal ganglion cell layer is thickest within the HAA from early embryonic stages.
- The HAA has lowest density of cones embryonically and highest density after hatching.
- Almost no apoptotic cell death was observed for photoreceptors in rod-free zone of HAA.
- Early specialization of the HAA suggests that there is a distinct progenitor pool for this area.
