# Supplementary material for: Characterization of the development of the high-acuity area of the chick retina
Source: Dev Biol. Author manuscript; Available in PMC 2025 Oct 10. (PMC12512175; doi:10.1016/j.ydbio.2024.03.005)

Supplementary Figure 1. Morphological development of the chicken HAA

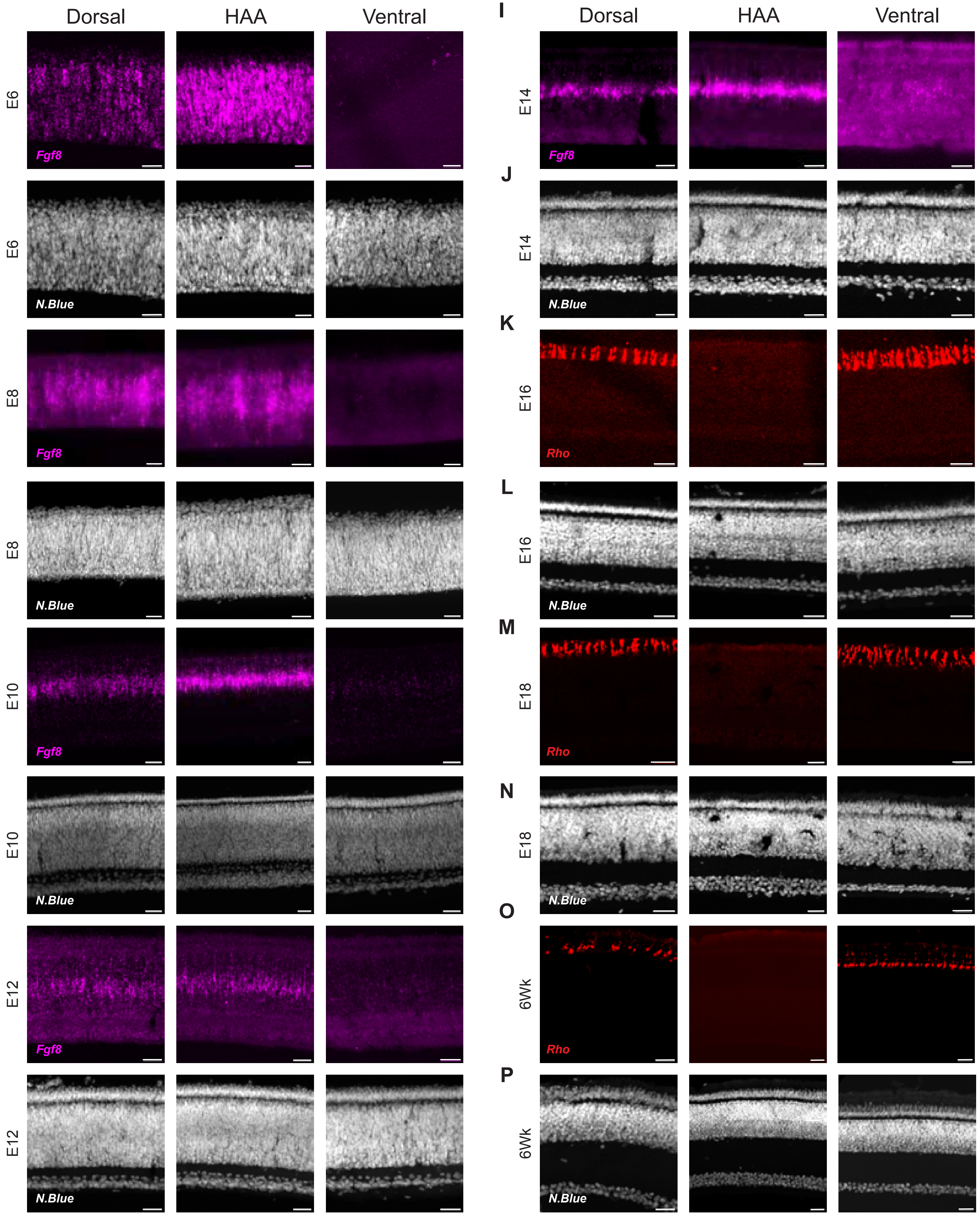

Supplementary Figure 2. Retinal thickness and apoptotic cell death during chicken development

A

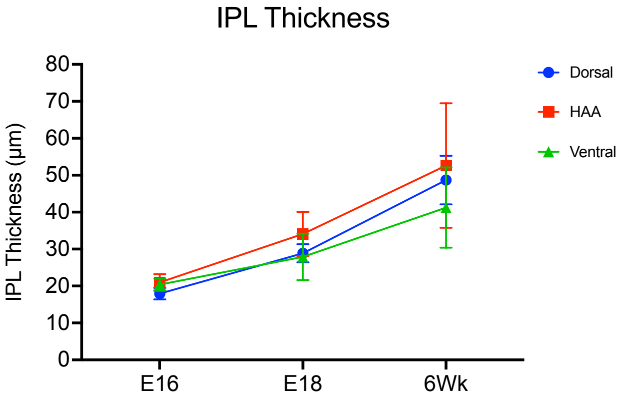

B

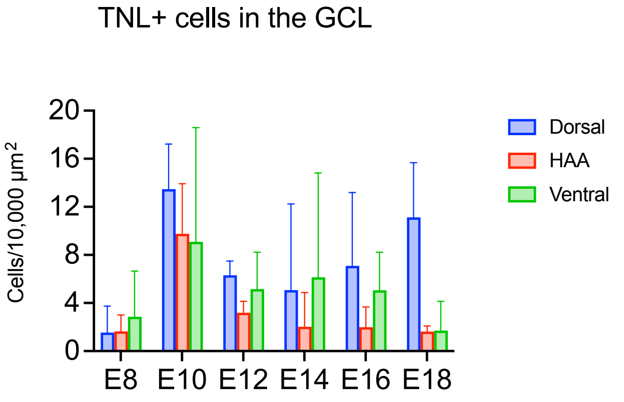

Supplementary Figure 3. Composition and densities of embryonic and adult opsins

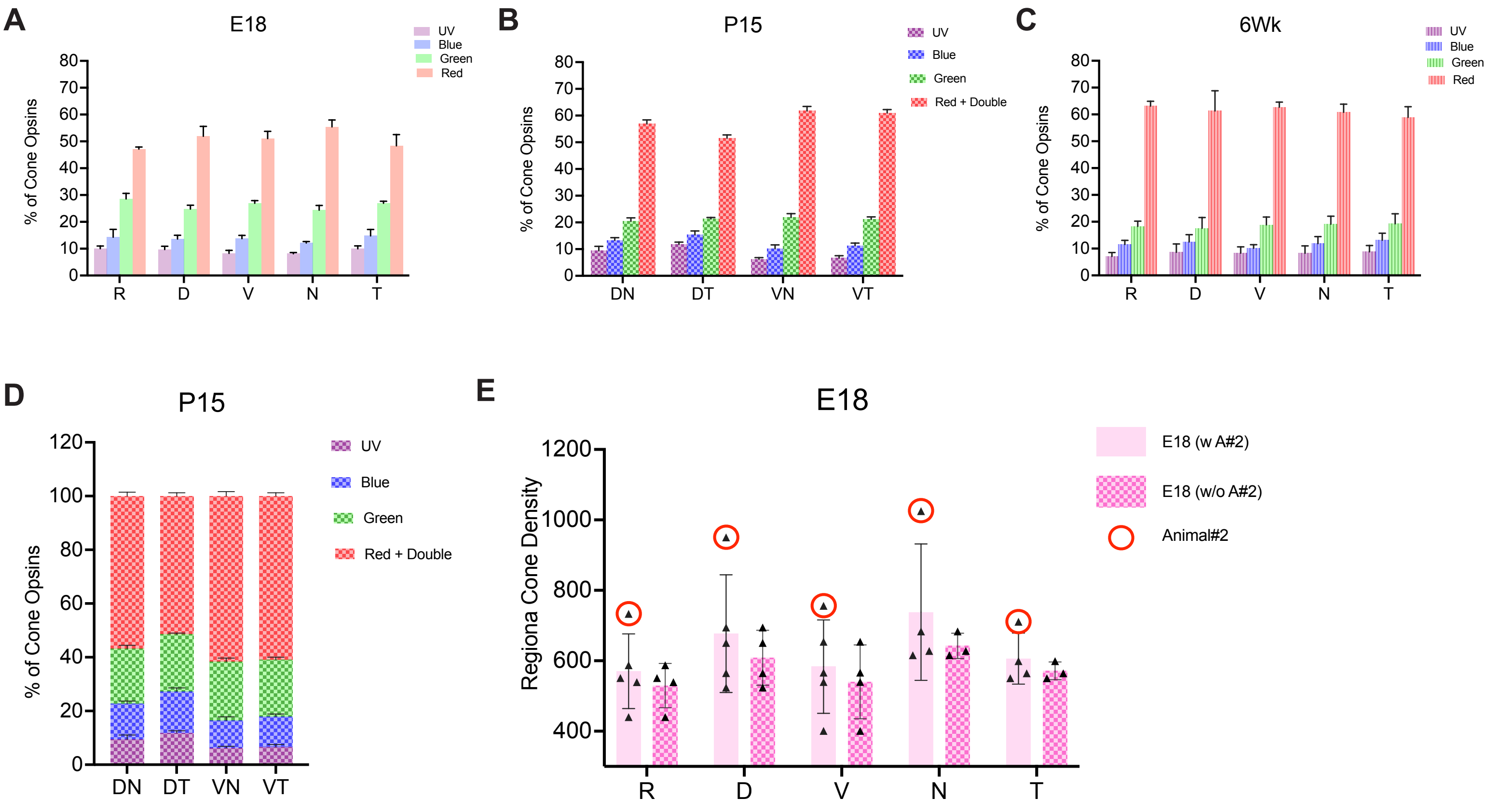

Supplement: MMC3 — Supplementary Figure 1. Morphological development of the chicken HAA. (A-P) Retinal cross-sections of the HAA, neighboring dorsal and ventral regions from E6 to 6Wks. The HAA was visualized in E6 to E14 retinas using HCR in situ hybridization for Fgf8 RNA (A, C, E, G, I), and for Rho RNA on E16, E18 and 6Wks retinas (K, M, O). Dorsal-HAA-Ventral sections were further stained with NucBlue to visualize retinal cell layers (B, D, F, H, J, L, N, P). Scale bar, 25 μm. Supplementary Figure 2. Retinal thickness and apoptotic cell death during chicken development. (A) Line graph comparing IPL thickness E16 to 6Wks within the HAA and neighboring dorsal and ventral regions in retinal cross-sections. Three retinas per timepoint were quantified. (B) Bar graph of number of TUNEL+ cells/10,000 μm2 in the GCL within the HAA and neighboring dorsal and ventral regions during chick retinal development. Three retinas per timepoint were quantified. IPL, Inner plexiform layer; GCL, Ganglion cell layer. Supplementary Figure 3. Composition and densities of embryonic and adult opsins (A, C) In situ HCR hybridization was conducted on retinal whole-mounts at E18 and 6Wks using probes for individual cone subtypes and rods. The regional composition of all cone photoreceptor subtypes is shown for E18 (A) and 6Wks (C) time points. (B, D) Cone opsin counts from P15 chicken retinas previously published (Kram et al. 2010) were reanalyzed to align with cone counting methods used in the current study. The regional composition of all cone photoreceptor subtypes is shown. (E) Regional cone photoreceptor density counts from E18 data, with and without Animal#2 which showed outlier values. R, RFZ; D, Dorsal; V, Ventral; N, Nasal; T, Temporal; DN, Dorsal-Nasal; DT, Dorsal-Temporal; VN, Ventral-Nasal; NT, Ventral-Temporal. [file NIHMS2072501-supplement-MMC3.pdf]
